# IMMEDIATE AND SUSTAINABLE EFFECTS OF TDCS ON PAIN IN OLDER ADULTS WITH ALZHEIMER’S DISEASE AND RELATED DEMENTIAS

**DOI:** 10.1093/geroni/igae098.3999

**Published:** 2024-12-31

**Authors:** Chiyoung Lee, Juyoung Park, Mindy Fain, James Galvin, Hyochol Ahn

**Affiliations:** University of Arizona, Tucson, Arizona, United States; University of Arizona, Tucson, Arizona, United States; University of Arizona, Tucson, Arizona, United States; University of Miami, Miami, Florida, United States; University of Arizona, Tucson, Arizona, United States

## Abstract

Chronic pain management is a complex clinical process requiring thorough assessment. Especially for older adults with Alzheimer’s disease and related dementias (ADRD), behavioral pain indicators are crucial in complementing self-reports. We simultaneously assessed the impact of transcranial direct current stimulation (tDCS) on mitigating both self-reported pain and pain behaviors to more objectively and comprehensively explore its effects in older adults with ADRD. The analysis investigated 40 participants randomly (1:1) subjected to active and sham tDCS for 20 min on 5 consecutive days. The numeric rating scale (NRS) and Mobilization–Observation–Behavior–Intensity–Dementia (MOBID-2) scale were used to assess self-reported pain and pain behaviors, respectively. Multi-group latent transition analysis enabled the simultaneous evaluation of both pain domains in a single model and analysis of their changes as a function of intervention exposure by modeling the transition probabilities of latent classes and comparing these changes between groups. Two latent pain categories (“high pain” and “low pain”) were identified based on NRS and MOBID-2 scores. Active group participants with “high pain” exhibited a 47.1% probability of transitioning to “low pain” immediately after intervention completion compared with no transition in the sham group. From immediate to 1-month follow-up, both groups exhibited positive transitions (44.3% vs. 39.2%). Active group participants with “high pain” at 1-month follow-up had a 58.4% likelihood of transitioning to “low pain” by 3 months post-intervention, whereas sham group participants exhibited no change. Our findings support the immediate and enduring effects of tDCS on pain reduction in older adults with ADRD.

